# Fleming’s Southern Hygienic Journal

**Published:** 1855-09

**Authors:** 


FLEMING'S SOUTHERN HYGIENIC JOURNAL; *Devoted exclusively to Human Health, edited by* NEWTON RANDOLPH FLEMING, M. D., *and published monthly at* $2 *per annum, in Atlanta, Georgia,*

Is the title of another candidate for public favor, and decidedly the most modest one that has come to our hands for many a day. The editor proposes striking "a blow for the physical regeneration of man," and also to make his journal "a newly risen star in the firmament of Science, whose mild beams shall cheer the million homes of America with the light of health and the hope of life." We sincerely trust that no one will be seriously hurt by Dr. Fleming's blow, and that astronomers will not be disturbed by the appearance of this new star in the Southern horizon.

